# Synchronous population dynamics in California butterflies explained by climatic forcing

**DOI:** 10.1098/rsos.170190

**Published:** 2017-07-19

**Authors:** Nicholas A. Pardikes, Joshua G. Harrison, Arthur M. Shapiro, Matthew L. Forister

**Affiliations:** 1 Program in Ecology, Evolution, and Conservation Biology, Department of Biology, University of Nevada, Reno, NV, USA; 2 Department of Biology, University of Nevada, Reno, NV, USA; 3 Center for Population Biology, University of California, Davis, CA, USA

**Keywords:** butterfly, ENSO, elevational gradient, population decline, spatial synchrony

## Abstract

A long-standing challenge for population biology has been to understand why some species are characterized by populations that fluctuate in size independently, while populations of other species fluctuate synchronously across space. The effects of climatic variation and dispersal have been invoked to explain synchronous population dynamics, however an understanding of the relative influence of these drivers in natural populations is lacking. Here we compare support for dispersal- versus climate-driven models of interspecific variation in synchrony using 27 years of observations of 65 butterfly species at 10 sites spanning 2750 m of elevation in Northern California. The degree of spatial synchrony exhibited by each butterfly species was used as a response in a unique approach that allowed us to investigate whether interspecific variation in response to climate or dispersal propensity was most predictive of interspecific variation in synchrony. We report that variation in sensitivity to climate explained 50% of interspecific variation in synchrony, whereas variation in dispersal propensity explained 23%. Sensitivity to the El Niño Southern Oscillation, a primary driver of regional climate, was the best predictor of synchrony. Combining sensitivity to climate and dispersal propensity into a single model did not greatly increase model performance, confirming the primacy of climatic sensitivity for driving spatial synchrony in butterflies. Finally, we uncovered a relationship between spatial synchrony and population decline that is consistent with theory, but small in magnitude, which suggests that the degree to which populations fluctuate in synchrony is of limited use for understanding the ongoing decline of the Northern California butterfly fauna.

## 1. Introduction

A primary goal of population ecologists is to understand the mechanisms that underlie fluctuations in the density of natural populations of plants and animals through both space and time. Early studies suggested that trophic interactions and exogenous forces, such as climatic variability, might play an important role in driving spatial and temporal population dynamics [1–3]. More recently, population biologists have integrated larger spatial and temporal datasets to describe the dynamics of spatially segregated populations. For example, metapopulation models are used to predict the persistence of subpopulations and understand drivers of metapopulation collapse [4,5]. A key parameter of interest in such studies is the extent to which subpopulations exhibit correlated spatio-temporal dynamics (e.g. experience ‘good years’ and ‘bad years’ in parallel) because this type of synchronization can limit the ability of metapopulations to recover from the loss of sub-populations [4–7]. Here we take a unique, multi-species approach using long-term data to advance understanding of correlated spatio-temporal dynamics in insect populations that exist across a heterogeneous landscape.

Three non-mutually exclusive mechanisms are often hypothesized to synchronize population dynamics among populations: (i) dispersal of individuals among populations, which links the dynamics of those populations; (ii) synchronization owing to density-independent factors (e.g. climate) that are correlated across wide areas (i.e. the ‘Moran effect’ [8]); and (iii) interactions with other species (e.g. natural enemies and pathogens) that are themselves either synchronous or highly mobile [9–11]. Identifying the relative influences of each of these three mechanisms is challenging because all three may cause similar patterns of synchrony among populations [10,12]. Moreover, it is difficult to directly measure the contribution of dispersal, which is itself a complex trait, and the product of other interacting biological characteristics [13]. Furthermore, data describing natural enemy population densities are not available for the majority of organisms, including our focal species; thus this investigation will focus on dispersal propensity and sensitivity to climatic variation, but not interspecific interactions.

**Figure 1.**
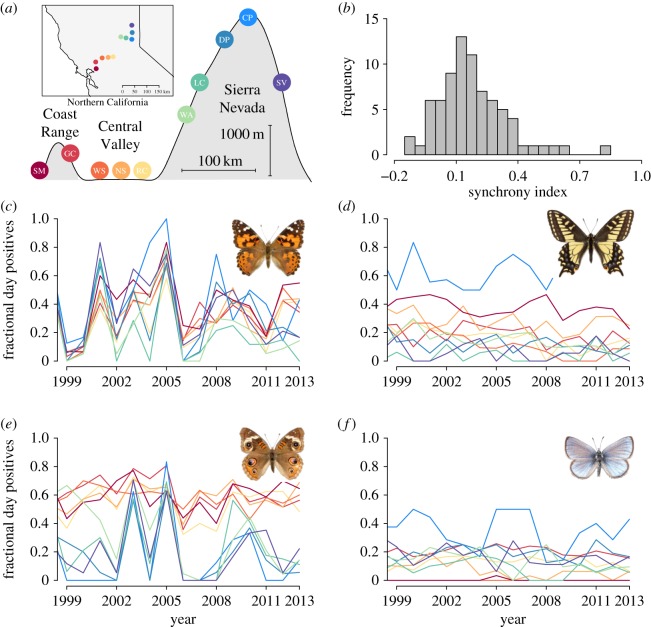
(*a*) Map of Northern California (inset) showing our 10 study sites, along with a portrayal of the elevational relief present. (*b*) Histogram displaying the frequency distribution of synchrony indices for the 65 butterfly taxa included in this study. (*c*–*f*) Time series (1999–2014) of four butterfly taxa representative of the variation in synchrony among species: (*c*) *Vanessa cardui* (synchrony index: 0.82); (*d*) *Papilio zelicaon* (index: 0.07); (*e*) *Junonia coenia* (index: 0.56); and (*f*) *Glaucopsyche lygdamus* (index: 0.09)). All photos used by permission from http://www.butterfliesofamerica.com/ (photographers: Kim Davis, Mike Strangeland, Andrew Warren).

To compare the relative influence of dispersal propensity and sensitivity to climatic variation on spatial synchrony, we used data from 27 years of observations collected by a single observer (A.M.S.) of 65 butterfly species across 10 sites that are separated by 210 km and span an elevational gradient of 2750 m (figure 1*a*). We characterized each butterfly species in terms of degree of spatial synchrony and a range of species-specific properties that together acted as an index of dispersal propensity, including: wingspan, geographical range, elevational range and host breadth (see Methods; [14–20]). We also quantified the sensitivity of each species to climatic variation (e.g. sensitivity to winter precipitation, summer temperature and other variables) using a hierarchical linear model implemented in a Bayesian framework. These data allowed us to compare several drivers of interspecific variation in spatial synchrony across all 65 butterfly species. We used structural equation modelling (SEM) to address the following questions: (i) is interspecific variation in spatial synchrony better predicted by dispersal propensity or sensitivity to climatic variation? (ii) can interspecific variation in spatial synchrony be modelled through the combined or interacting effects of dispersal propensity and climatic sensitivity? Finally, given the theoretical expectation that synchrony can predispose metapopulations to collapse, we ask if an improved understanding of the drivers of spatial synchrony can shed light on declines in focal butterfly populations [5,21–23]. The portion of Northern California where our study sites are located has been characterized by dramatic population declines and local extirpations of butterfly taxa in recent years, particularly at low elevations [24]. These declines have been attributed to a combination of development, changing land use and pesticides [25,26], but the contribution of spatial synchrony to these declines has not been studied.

## 2. Methods

### 2.1 Study system, data robustness and calculation of synchrony

Butterfly data were collected by A.M.S. at 10 locations in Northern California from 1972–2013 (figure 1*a*). These sites include a variety of habitat types, spanning a 2750 m elevational gradient, and are separated by 210 km from the most western to the most eastern location. A fixed transect was walked every two weeks as per [27], and incidence of taxa noted (for maps of transects see http://butterfly.ucdavis.edu/). Surveys were conducted in spring, summer and autumn on sunny days with little wind, and thus suitable for butterfly flight. From these records, we calculate a fraction of days per year in which a species was seen at a particular site (specifically, the number of positive observations was divided by the number of visits to account for variation in sampling effort; henceforth this index is referred to as fractional day positives (FDP)). Previous analyses have shown that variation in FDP effectively represents variation in butterfly abundance [28]. To confirm the previous finding, our primary analyses (described below) were repeated using count data for a subset of taxa and sites (those in the central valley of California only) for which counts of individuals were available. In all cases, results obtained with count data were similar to results obtained with occurrence data, despite counts of abundance being inherently more variable than incidence records. Considering the congruence of results with incidence and abundance data, and the fact that a single observer collected all data across years and sites, we infer that the results reported here are robust to variation in detectability, which (similar to many other insect monitoring programmes) has not been separately quantified in our system. Moreover, analyses were repeated while omitting observations for randomly selected taxa (see below), and results were similar; confirming that variation in detectability among taxa has not confounded our analyses.

For each combination of site and taxon, the previous year's FDP was subtracted from the current year's FDP to calculate a change in FDP between years (ΔFDP). Correlation coefficients were then calculated between ΔFDPs from different sites in a pairwise fashion (Pearson's *r*), as has been done with a variety of taxa in studies of spatial synchrony [9,29]. For each pairwise correlation, data from the sites with the longer histories of observation were truncated to match the site with the shortest record (all sites considered had at least 25 years of observations). The resulting correlation coefficients were averaged across all pairwise comparisons among sites to give a taxon-specific index of synchrony. If a species was absent for eight or more years at a site, then that site was not included in the analysis for that species. Finally, for a species to be included in this study it had to occur at three or more sites. In total, synchrony indices for 65 butterfly species were generated (electronic supplementary material, table S1).

### 2.2. Structural equation models

To compare specific hypotheses for the drivers of synchrony, we used SEM. This method facilitates the testing of causal relationships among variables*,* including comparison of direct and indirect causal structures [30]. A total of six SEMs were constructed to compare *a priori* hypotheses about potential drivers of spatial synchrony, based on insights gained from previous work with these butterflies and sites [24,31–33]. Two SEMs were generated to independently compare the influence of dispersal propensity and sensitivity to climatic variation on synchrony, and a third SEM was assembled to investigate the combined influence of both drivers. For each species, the average number of sites occupied (to account for variation in number of time series available to calculate synchrony indices), the average FDP across sites (‘abundance’; to account for variation in population density), and the average inter-annual change in FDP across sites (henceforth ‘trend’, as a measure of population increase or decrease, see Forister *et al*. 2010 [24]) were calculated, *z*-standardized, and included as covariates in SEMs to account for their influence on spatial synchrony. The inclusion of trend in each model allowed us to quantify the association between interspecific variation in synchrony and rate of inter-annual population change (for most species, populations were in decline).

We also considered including maximum distance between sampling sites as a covariate, but found that it was highly correlated with average number of sites occupied (Pearson's correlation = 0.76); suggesting that average number of sites occupied served as a good proxy for distance between sites. Furthermore, the incorporation of maximum distance between sites did not improve model performance or change the overall conclusions drawn from each model; therefore we did not include maximum distance between sampling sites in SEMs.

For all models, we further investigated the influence of dispersal by removing nine butterfly species that undergo annual migrations (including latitudinal and elevational migrations) and observed changes in model fit and path coefficients (electronic supplementary material, table S1). Since these migratory taxa are known to be extreme dispersers, they represent a subset of species whose variation in synchrony is likely to be influenced by traits predictive of dispersal propensity and thus their removal can provide an informative contrast to analyses solely encompassing more sedentary butterflies. To understand how the removal of nine species from our analysis affected the variance explained, each SEM was performed 1000 times with a random set of 56 butterfly species (dropping nine each time). The mean and standard error of variance explained were calculated for each separate model (e.g. dispersal, climate, combined). Details of SEM construction are provided below. Model fit was assessed using *χ*^2^, and model comparison performed using the Akaike information criterion (AIC) [34]. All SEM and path analyses were constructed using the lavaan package v.0.5-17 [35] in R v.3.1.1 [36].

### 2.3. Modelling the influence of dispersal propensity on synchrony

Given that dispersal is difficult to quantify and often comprised of several variables, a maximum-likelihood factor analysis was used to reduce the dimensionality among correlated data that together characterize dispersal propensity among taxa (R package: psych v.1.4.8.11; [37]). Wingspan, geographical range, diet breadth (number of plant genera consumed), and elevational range were selected to represent dispersal propensity within this butterfly assemblage. These variables were chosen because interspecific variation in butterfly dispersal ability has been linked to wingspan (e.g. [17,19,20], geographical range and diet breadth [14–16,18]. Geographical range (km^2^) for each taxon was taken from [38,39] and diet breadth was taken from [40]. Diet breadth included only those larval hosts used in Northern California. Wingspan was taken from [41] and was the mean value of the range reported for each species.

Two factors were calculated that respectively explained 30% (‘Dispersal 1’) and 15% (‘Dispersal 2’) of the variance in underlying variables. ‘Dispersal 1’ included all four variables, but was most heavily weighted by geographical range, diet breadth and elevational range. ‘Dispersal 2’ included all variables except diet breadth, and was primarily associated with wingspan and geographical range (see the electronic supplementary material, tables S2 and S5 for loadings). These two factors were input into SEMs and served as latent variables. Latent variables are used to model unobservable, or highly multidimensional phenomena (e.g. dispersal propensity) using information from more easily measurable phenomena (e.g. wingspan, geographical range).

### 2.4. Modelling response to weather

A hierarchical Bayesian linear modelling framework was used to model the response to climatic variation by each butterfly species. This approach allowed us to account for the hierarchical structure within our data (i.e. sites nested within transect) and to leverage information from those sites with more occurrences for a given taxon when determining parameter estimates for sites with fewer occurrences. These responses were subsequently used during calculation of factors characterizing variation in sensitivity to climate among taxa (see below). Climate information was extracted from the PRISM dataset [31], which interpolates data from weather stations with respect to site-specific topography. Data were converted to seasonal values following a ‘water year’ format, so that spring consisted of March, April, and May; summer of June, July, and August; autumn of September, October, and November of the previous year; and, winter of December of the previous year, and January and February of the current year. This ‘water year’ corresponds to the post-summer increase in precipitation typically observed beginning in September through much of Northern California. Prior to model construction, all seasonal weather variables were converted to *z*-scores. To identify responses to the El Niño Southern Oscillation (ENSO; a primary driver of long-term natural climatic variation in Northern California [42]) we used the sea-surface temperature anomaly (SSTA) dataset from 1981–2010 in the ‘Niño 3.4’ region of the Pacific Ocean (Climate Prediction Center of the National Oceanographic and Atmospheric Administration). The SSTA is defined as a departure from the long-term SST mean, and is a commonly used index of the strength of ENSO. The mean values of SSTA of December, January, and February from a given ‘water-year’ were used in analyses because they correspond to the peak of ENSO [43]. All weather variables were chosen because previous work has shown the response to these weather conditions to be important drivers of butterfly population dynamics in Northern California [31–33]. The average pairwise correlation of principle components from a principle component analysis of all weather variables considered in this study was 0.82. In addition, year was included as a covariate in each model to describe inter-annual trends in population density [24].

For each taxon, a binomial response consisting of day positives and visits was modelled. This response was linked to the predictor variables of a hierarchical linear model using an inverse logit link function: $p_{ij}=1\text{/}(1+{\text{e}}^{-\alpha_{ij}})$, where *p_ij_* is the proportion of occurrences out of total visits in year *i* and at site *j*, and *α_ij_* is the output of the linear model for year *i* at site *j*. The linear model was of the form:

$$\begin{array}{rl} \alpha_{ij} & =\mu_{j}+\beta_{1j}{\text{ winter temp}}_{ij}+\beta_{2j}{\text{ spring temp}}_{ij}+\beta_{3j}{\text{ winter precip}}_{ij}\\ & \;+\beta_{4j}{\text{ spring precip}}_{ij}+\beta_{5j}{\text{ summer precip}}_{ij}+\beta_{6j}{\text{SSTA}}_{ij}+\beta_{7j}{\text{ year}}_{ij}. \end{array}$$

The mean estimate of FDPs for a given taxon at a given site is given by the intercept term *μ*, and regression coefficients for each model term by *β*_1–7_. Normal distributions with means and precisions equal to transect-wide parameters were used as sampling pools for site-specific intercepts and beta coefficients:

$$\mu_{j}∼N(\mu_{\mu },\tau_{\mu })$$

and

$$\beta_{Kj}∼N(\mu_{\beta_{K}}\tau_{\beta_{K}}),$$

where *k* is the number associated with each model term. We used uninformative hyperpriors for these parameters defined by:

$$\begin{array}{rl} \mu_{\mu } & ∼N(0,1.0{\text{e}}^{-5}),\\ \mu_{\beta_{K}} & ∼N(0,1.0{\text{e}}^{-5}),\\ \tau_{\mu } & ∼\text{Gamma}(0.1,1.0{\text{e}}^{-3})\\ \;\text{and}\;\;\tau_{\beta_{K}} & ∼\text{Gamma(}0.1,1.0{\text{e}}^{-3}\text{)}. \end{array}$$

Posterior probability distributions (PPDs) for the transect-wide impact of each model term were approximated via Markov chain Monte Carlo sampling using rjags (v.3.4.0, [44]). Two sampling chains were run for 30 000 iterations following a burn-in of 1000 iterations. Effective sample sizes and trace plots were examined to ensure adequate mixing and convergence on a suitable approximation of PPDs. The mean of the PPD for the transect-wide estimate of each regression coefficient was used as an estimate of the response to that term. The outputs of this approach were estimates of species-specific responses to weather variables that were informed by responses across all study sites (electronic supplementary material, table S1).

### 2.5. Modelling the influence of climate on synchrony

A maximum-likelihood factor analysis was used to reduce the dimensionality of data describing how taxa respond to climatic variation as output from hierarchical linear modelling described above [37]. We calculated two factors from the analysis of responses to precipitation in spring, summer, and winter and temperature in spring and winter, which explained 28% and 21% respectively of the variance in analysed variables. We included ENSO (as measured by response to SSTA; see above) into our SEM as a standalone variable to compare the influence of regional climate versus local weather on synchrony, and therefore sensitivity to ENSO was not included in the factor analysis. Factor one (‘Climate 1’) was composed of responses to all five climatic variables, but was most heavily weighted by spring temperature and to a lesser degree, spring precipitation. Factor two (‘Climate 2’) included all climate variables except response to summer temperature, and was primarily weighted by responses to winter temperature (electronic supplementary material, tables S3 and S4).

### 2.6 Modelling the combined influence of natural history and sensitivity to weather on synchrony

We also examined the combined influence of variation in dispersal propensity and sensitivity to weather on spatial synchrony via SEM. Both sets of latent variables used in the previous analyses were included in our ‘combined’ model. This allowed us to compare the relative influence of sensitivity to weather and dispersal propensity on synchrony in the same model. We hypothesized *a priori* that dispersal propensity and sensitivity to climate might interact to influence spatial synchrony, therefore we generated models linking the latent variables characterizing both of these drivers. We compared performance among models (using AIC and *χ*^2^) to determine which combination of latent variables improved model fit.

## 3. Results

Our index of spatial synchrony, which measures the correlation of changes in yearly abundances across populations [9], identified 44 out of the 65 butterfly species as having synchrony indices greater than 0.1; only seven taxa had negative synchrony indices, which indicated asynchronous fluctuations (minimum index value was −0.11) (figure 1*b*). By visual inspection, index values greater than 0.2 represented fairly synchronized population dynamics, and values greater than 0.4 highly synchronized dynamics (for examples see figure 1*b–f*). Out of all species studied, 25 had synchronized dynamics (more than 0.2) and five species had indices over 0.4 (electronic supplementary material, table S1).

**Figure 2.**
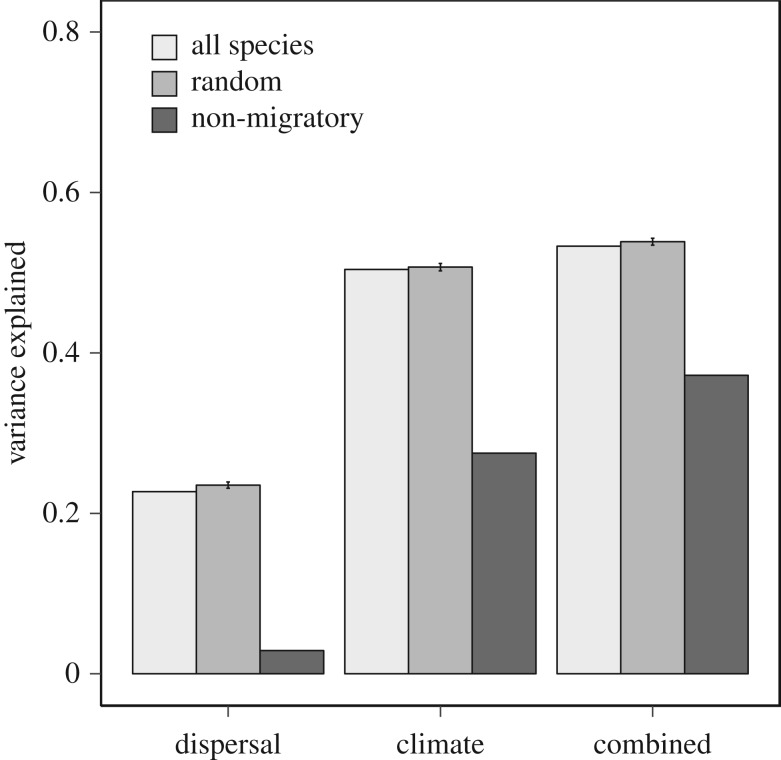
Explained variance in spatial synchrony among Northern California butterfly species by SEM: limited to dispersal propensity (‘dispersal’), sensitivity to climate (‘climate’), or the combined influences of both drivers of spatial synchrony (e.g. dispersal and climate) (‘combined’). Dark grey bars represent SEM models from which migrants were excluded (leaving 56 species), while light grey bars represent variance explained when all species were considered (65 species). The ‘random’ bar represents variance explained for each model when nine species were randomly removed from the original 65 species. Models were permuted 1000 times and the mean and 95% confidence interval of variance explained is plotted (see Methods).

**Figure 3.**
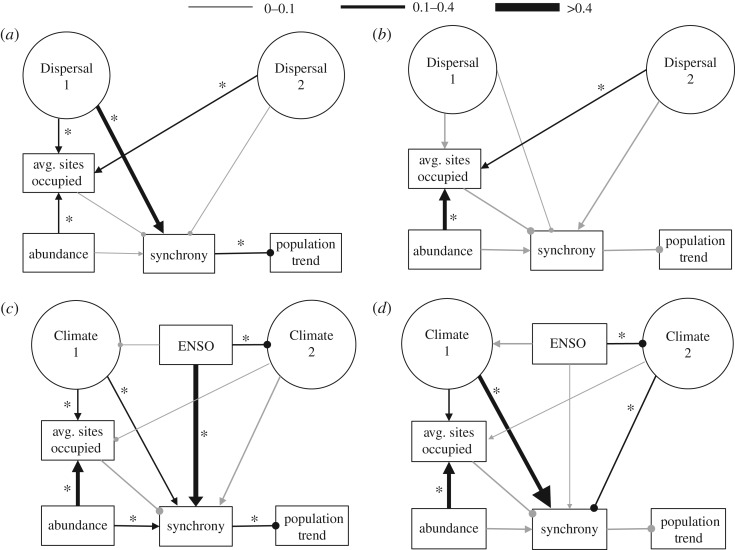
Structural equation models (SEM) of synchrony as driven by taxon-specific responses to climatic variation and natural history traits associated with dispersal. Circles represent ‘latent’ variables generated by factor analysis that together describe a taxon's sensitivity to local weather conditions and natural history traits associated with dispersal (see Methods). The variable denoted ‘abundance’ is the average fraction of days a species was observed (out of the total number of visits per year) across the study area over the 27 year long study period—a proxy for abundance [28]. The variable denoted ‘avg. sites occupied’ is the average number of sites occupied by a taxon across the study area over the study period. ‘Population trend’ refers to inter-annual trend in population density for a particular taxon. In all three models, path coefficients were standardized and path widths scale with coefficient sizes (see legend at top of figure). Arrows represent positive coefficients, while lines ending with a circle represent negative coefficients. Paths in grey represent insignificant coefficients, while those in black with an asterisk (*) denote significance (*p* ≤ 0.05; see supplementary tables for exact *p*-values). Model fit was determined using a *χ*^2^ test; non-significant *p* values denote a supported model. (*a*) ‘Natural history’ SEM modelling synchrony as driven by natural history traits with all butterfly species included ($\chi_{4}^{2}=1.41$, *p* *=* 0.84, *n* = 65). (*b*) ‘Natural History’ SEM modelling synchrony as driven by natural history with migratory butterfly species excluded ($\chi_{4}^{2}=4.42$, *p* *=* 0.35, *n* = 56). (*c*) ‘Climate’ SEM to model synchrony as driven by sensitivity to climate with all butterflies included ($\chi_{9}^{2}=5.41$, *p* *=* 0.80, *n* = 65). ENSO refers to sensitivity of a taxon to the sea surface temperature anomaly, a proxy for the severity of the El Niño Southern Oscillation (ENSO). (*d*) ‘Climate’ SEM to model synchrony as driven by sensitivity to climate with migratory butterflies excluded ($\chi_{9}^{2}=16.36$, *p* *=* 0.06, *n* = 56; see the electronic supplementary material, table S1 for which species were excluded).

Our models successfully explained variation in spatial synchrony among Northern California butterflies (figure 2). We confirmed the contribution of dispersal propensity to interspecific variation in spatial synchrony using SEM (figure 3*a*; $\chi_{4}^{2}=1.41$, *p* *=* 0.84, *n* = 65; higher *p*-values signify better fit; electronic supplementary material, table S6). This SEM explained 23% of the variance in spatial synchrony among taxa, and 59% of the variation in the average number of sites occupied across the elevational gradient. The latter result suggests that our latent variables captured meaningful biological variation pertaining to dispersal ability. The influence of dispersal propensity on patterns of spatial synchrony was restricted to the positive influence of a single latent variable (‘Dispersal 1’), which was primarily weighted by diet breadth and geographical range. Removing migratory species from the SEM reduced the explanatory power of the model (figure 3*b*; $\chi_{4}^{2}=4.42$, *p* *=* 0.35, *n* = 56; electronic supplementary material, table S7), which subsequently only explained 3% of the variance in spatial synchrony.

Our ‘climate’ SEM was well supported, and revealed that sensitivity to climate, especially to the large-scale climate pattern ENSO, was strongly, positively associated with variation in spatial synchrony among butterfly taxa (figure 3*c*; $\chi_{9}^{2}=5.41$, *p* *=* 0.80, *n* = 65; electronic supplementary material, table S8). This SEM explained 50% of the variation in spatial synchrony among butterflies. ENSO drives regional climate patterns and the response to ENSO was the strongest predictor of spatial synchrony for the entire fauna, with butterflies more sensitive to ENSO exhibiting greater synchrony. We also observed that those butterfly species most responsive to ENSO were less responsive to local climatic conditions.

When excluding migratory butterflies, which are especially sensitive to ENSO fluctuations [33,43], SEM performance decreased (figure 3*d*; $\chi_{9}^{2}=16.36$, *p* *=* 0.06, *n* = 56; electronic supplementary material, table S9) and the role of ENSO as a driver of spatial synchrony was diminished. This is consistent with the previously-observed importance of regional weather for the most dispersive and widespread species [33]. However, model performance was still high and explained 28% of the variance in spatial synchrony among species. Sensitivity to local weather was the best predictor of variation in synchrony for non-migratory butterflies. Species with the most asynchronous dynamics were also the most sensitive to local weather, in particular spring and summer precipitation.

**Figure 4.**
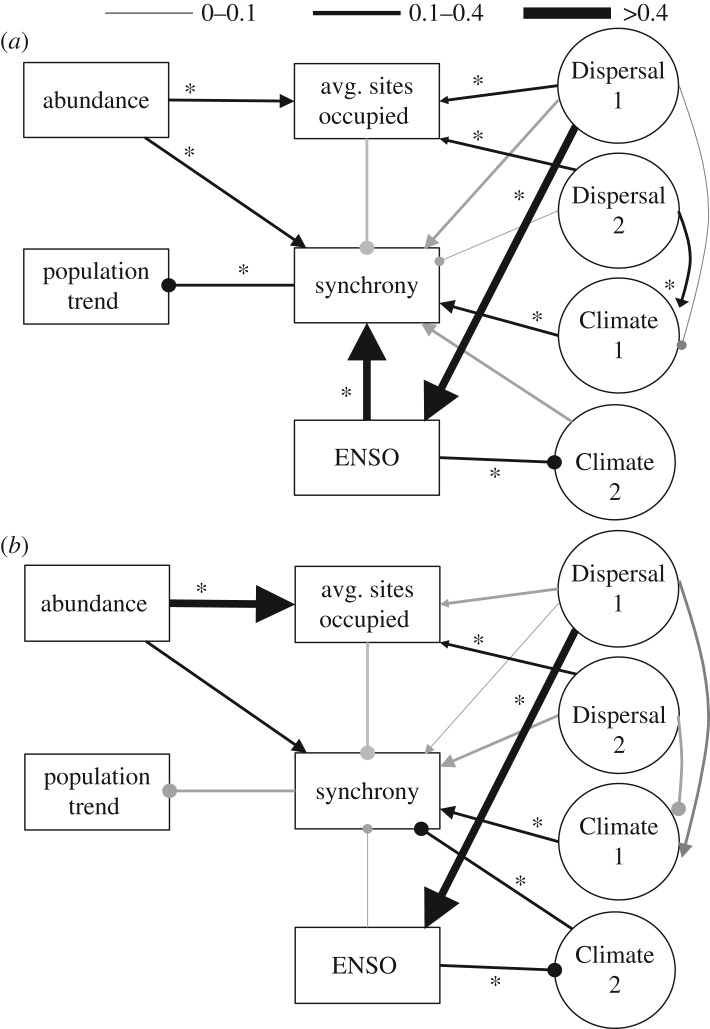
Structural equation models (SEM) that describe the combined effects of natural history traits and sensitivity to weather on spatial synchrony. Paths are represented similarly to figure 3. ‘Dispersal 1’ and ‘Dispersal 2’ refer to two factors extracted from a factor analysis of species-specific dispersal propensity (see Methods). ‘Climate 1’ and ‘Climate 2’ refer to two factors extracted from a factor analysis of sensitivity to local weather. (*a*) An SEM constructed using data from all focal species, which was well supported ($\chi_{18}^{2}=15.2$, *p* *=* 0.65, *n* = 65). (*b*) An SEM calculated while omitting migratory taxa (electronic supplementary material, table S1) which was not supported ($\chi_{18}^{2}=36.1$, *p* *=* 0.007, *n* = 56), though individual path coefficients remain informative.

The ‘combined’ SEM, which included both dispersal propensity and climatic sensitivity, was also strongly supported and explained 53% of the variance associated with spatial synchrony among species (figure 4*a*; $\chi_{18}^{2}=15.19$, *p* *=* 0.65, *n* = 65; electronic supplementary material, table S10). In line with results from our climate SEM, sensitivity to climatic variation was the best predictor of spatial synchrony, and both sensitivity to local weather and ENSO resulted in more synchronous dynamics among butterflies; with sensitivity to ENSO being the strongest predictor of synchrony. A significant, direct influence of dispersal on spatial synchrony was not observed, but we did uncover several indirect effects of dispersal mediated by sensitivity to climate (figure 4*a*). Both indirect effects of dispersal propensity positively influenced spatial synchrony, and provide evidence that the role of dispersal propensity on synchrony is probably mediated by climate. Repeating the ‘combined’ SEM without migratory butterflies resulted in an unsupported causal structure (figure 4*b*; $\chi_{18}^{2}=36.05$, *p* *=* 0.01, *n* = 56; electronic supplementary material, table S11). However, path coefficients were still informative because they represent the output of pairwise regression, and the model explained 35% of the variation in synchrony associated with non-migratory butterflies. Without migratory species, the direct influence of sensitivity to ENSO on synchrony was lessened and an indirect influence of ENSO on synchrony, via local weather, became evident. In all models, the variance explained when nine random species was removed was equal to models that included migratory species, supporting the idea that the nine migratory species are biologically unique among this butterfly assemblage (figure 2).

Finally, we considered the effect of spatial synchrony on trends in inter-annual population change (figures 3 and 4; electronic supplementary material, tables S6–S11). For each SEM, synchrony explained only approximately 5% of the variation in inter-annual trend across taxa. However, in all three SEMs the direct path coefficient from spatial synchrony to population trend was significant (*p* < 0.05). The strength and significance of this path depended on the incidence of migratory species in the model. Removing migratory species eliminated the path's significance and narrowly reduced the strength of the coefficient in all three cases. In all three models, a negative coefficient was observed, suggesting that higher levels of spatial synchrony are associated with population declines among the butterfly assemblage, particularly for migratory species.

## 4. Discussion

In this study we identified relationships between spatial synchrony and both dispersal propensity and sensitivity to climatic variation, among 65 butterfly species in a region characterized by extreme habitat heterogeneity (figure 1). Our approach differs significantly from previous investigations that focused on correlations between climatic fluctuation and population dynamics in that we linked species-specific climatic sensitivity and dispersal propensity to the degree of synchrony exhibited [45,46]. We have shown that the majority of interspecific variation in spatial synchrony can be explained through sensitivity to climatic variation, especially to large-scale climate patterns such as ENSO, the effect of which is at least partially mediated by traits related to dispersal.

In the portion of California where our transect is located, ENSO may lead to either increased or reduced precipitation, but effects on precipitation are dramatic [42]. Our results support previous efforts which have shown that large-scale climate patterns can act to synchronize population dynamics across entire regions [47,48]. The importance of sensitivity to ENSO was driven by the inclusion of migratory species in models. Indeed, removal of these taxa (*n* = 9) reduced the explained variance of models and dramatically altered the strength and direction of associated standardized path coefficients. A possible explanation for this is that the population dynamics of migratory species are shaped by climate across a broader spatial scale than more sedentary species.

By contrast, variation in spatial synchrony among non-migratory species was best predicted by sensitivity to localized weather conditions (species that are most sensitive to local weather have the least synchronized dynamics). Given the elevational range encompassed in this study and the corresponding breadth of habitat types, the influence of local weather conditions on butterflies may vary between sites, which may act to desynchronize sub-populations of conspecifics occurring across the transect. Indeed, previous investigations have shown that butterfly species can differentially respond to the same climatic variable (e.g. winter precipitation) at different sites [33].

Interestingly, sensitivity to spring and summer precipitation was indicative of taxa with asynchronous dynamics, and sensitivity to spring temperature and winter precipitation was representative of taxa with synchronous dynamics. These results complement previous work showing that species with highly fluctuating population dynamics are positively influenced by increased spring and summer precipitation, and negatively influenced by increasing spring temperatures and winter precipitation [32]. Moreover, drier winter conditions have previously been linked to earlier emergence time in California butterflies [49] while increased winter precipitation has a generally positively influence on the abundance of butterflies in the region [31]. When taken together, these results suggest that spatial synchrony for non-migratory taxa is associated with the ability to rapidly increase in abundance under suitable climate conditions.

Dispersal is thought to be an important contributor to spatial synchrony, yet with the exception of known migrants, species-specific dispersal propensity was not a strong predictor of interspecific variation in spatial synchrony (figure 2). Work with other Lepidoptera species also reports that dispersal plays a minor role in synchronizing populations [50–52]. A possible explanation for why increased dispersal propensity did not increase spatial synchrony is that non-migratory butterflies (the majority of species we examined) may rarely move between sites. Our focal sites span 2750 m of elevation and many habitat types, thus habitat heterogeneity may limit effective dispersal between disparate sites. This hypothesis is supported by a decline in variance explained when migratory species were omitted from our model (from 23% to 3% variance explained). This suggests that dispersal can indeed act as a synchronizing influence, but only for the most mobile taxa. Additionally, we acknowledge that interactions with natural enemies [53–55] probably account for a portion of the unexplained variance in our models of spatial synchrony. However, we were unable to assay the influence of natural enemies because relevant information was unavailable for even a subset of our focal taxa.

The abundances of most butterfly species occurring at lower elevations in our study area are in decline [24]. We detected these declines using our ‘trend’ index, which measures the inter-annual rate of population decline for each butterfly species averaged across the entire study area. Theory predicts that spatial synchrony within a metapopulation will be related to extinction propensity of the entire metapopulation [22], and there are several mechanisms that potentially link these phenomena. For example, spatially synchronous dynamics can reduce the beneficial effect of dispersal by making it less likely that populations experiencing a bad year are rescued by populations in a productive year [56]. Consistent with theory, we detected a significant negative association of increased spatial synchrony with population trends, such that more synchronized species were characterized by more severe declines in abundance over the course of the study. Note, however, that our measure of decline is a measure of population density averaged across focal sites, not a measure of metapopulation occupancy. Thus the mechanistic connection between synchrony and declining populations at our sites will have to wait on future studies potentially involving regional population surveys beyond our focal sites.

## 5. Conclusion

We report that interspecific variation in spatial synchrony among the butterflies of Northern California is best explained by sensitivity to climatic variation. Sensitivity to the large-scale climate pattern, ENSO was highly predictive of spatial synchrony, particularly so for the most mobile species (migrants). Dispersal propensity was less predictive of spatial synchrony than climate, especially for non-migratory species. However, our analyses revealed that both drivers influenced the degree of synchrony exhibited by butterflies. Finally, spatial synchrony appears to contribute little to the ongoing declines in butterfly abundance in this assemblage. In a world ever more characterized by habitat fragmentation, climate change, and consequent sub-division of populations, understanding the forces that drive variation in spatial synchrony among species is critical if we wish to understand natural populations and establish a baseline against which future changes can be measured.

## Supplementary Material

Electronic Supplementary Material

## Supplementary Material

Supplemental Data

## Data accessibility

Raw data of butterfly observations are available from http://butterfly.ucdavis.edu/. Processed data used for analyses reported in this manuscript are included as ‘Supplemental Data’ in the electronic supplementary material associated with this article.
