# Supplementary material for: Synchronous population dynamics in California butterflies explained by climatic forcing
Source: R Soc Open Sci. 2017 Jul 19;4(7):170190. doi: 10.1098/rsos.170190 (PMC5541541; doi:10.1098/rsos.170190)
Supplement: Electronic Supplementary Material [file rsos170190supp1.docx]

Supplemental Tables and Figures

| Table S1: Butterfly taxa included in this study arranged in order of decreasing synchrony. Asterisks (*) denote migratory taxa (including both altitudinal and latitudinal migrants). “Synch” denotes spatial synchronicity index, with higher values indicating more synchronous taxa (see *Materials and Methods*). “Trend” is the mean of a posterior probability distribution (PPD) of the standardized partial regression coefficient of the effect of year on fractional day positives (see *Materials and Methods* for model details, including covariates). “ENSO” refers to the mean of a PPD of the standardized partial regression coefficient for the sea surface temperature anomaly (SSTA), which is a measure of the severity of the El Niño Southern Oscillation (ENSO). “Sites Occ.” refers to the number of sites occupied by a given taxon each year averaged across all years. “Avg. FDP” refers to the average fractional day positives of a given taxon across sites and years; it is a proxy for average yearly abundance. | | | | | | |
| --- | --- | --- | --- | --- | --- | --- |
| Species | Synch. | Trend | ENSO | Sites Occ. | | Avg. FDP |
| *Vanessa cardui** | 0.82 | 0.06 | 0.51 | 9.23 | 0.35 | |
| *Pontia protodice** | 0.60 | -0.88 | 0.55 | 6.23 | 0.16 | |
| *Junonia coenia** | 0.56 | -0.20 | 0.17 | 8.88 | 0.43 | |
| *Lerodea eufala* | 0.45 | -0.75 | -0.04 | 4.62 | 0.14 | |
| *Vanessa annabella** | 0.42 | -1.01 | 0.04 | 9.46 | 0.43 | |
| *Atalopedes campestris* | 0.37 | -0.37 | 0.10 | 6.23 | 0.22 | |
| *Lycaena editha* | 0.37 | 0.02 | -0.06 | 3.31 | 0.26 | |
| *Pyrgus scriptura* | 0.36 | -0.88 | 0.03 | 2.92 | 0.27 | |
| *Danaus plexippus** | 0.35 | -0.72 | -0.01 | 9.08 | 0.30 | |
| *Vanessa atalanta** | 0.35 | -0.31 | 0.07 | 6.92 | 0.18 | |
| *Phyciodes mylitta* | 0.34 | -0.21 | -0.02 | 9.50 | 0.43 | |
| *Pontia occidentalis* | 0.32 | -0.51 | -0.07 | 3.23 | 0.38 | |
| *Erynnis tristis* | 0.31 | -0.24 | -0.07 | 5.73 | 0.16 | |
| *Brephidium exile* | 0.30 | -0.46 | 0.03 | 5.46 | 0.15 | |
| *Poanes melane* | 0.29 | -0.19 | -0.12 | 4.04 | 0.08 | |
| *Celastrina ladon echo* | 0.28 | -0.38 | 0.03 | 5.85 | 0.17 | |
| *Lycaena xanthoides* | 0.28 | -0.66 | 0.04 | 3.35 | 0.08 | |
| *Atlides halesus* | 0.27 | -0.31 | 0.03 | 5.27 | 0.05 | |
| *Hylephila phyleus* | 0.26 | -0.26 | -0.01 | 6.15 | 0.27 | |
| *Euphydryas chalcedona* | 0.24 | -0.36 | 0.00 | 4.15 | 0.09 | |
| *Nymphalis antiopa** | 0.22 | -0.64 | -0.10 | 9.15 | 0.25 | |
| *Colias eurytheme** | 0.21 | -0.24 | 0.03 | 10.00 | 0.74 | |
| *Hesperia juba* | 0.21 | -0.20 | -0.08 | 4.65 | 0.30 | |
| *Euphilotes battoides* | 0.20 | 0.25 | -0.04 | 3.38 | 0.12 | |
| *Pyrgus communis* | 0.20 | -0.18 | -0.01 | 9.92 | 0.53 | |
| *Erynnis persius* | 0.20 | -0.14 | 0.05 | 4.27 | 0.07 | |
| *Ochlodes agricola* | 0.20 | -0.09 | 0.01 | 2.65 | 0.11 | |
| *Lycaena helloides* | 0.20 | -0.43 | -0.02 | 6.88 | 0.21 | |
| *Papilio eurymedon* | 0.18 | -0.06 | -0.01 | 6.00 | 0.18 | |
| *Pieris napi* | 0.17 | -0.10 | 0.06 | 3.08 | 0.19 | |
| *Euchloe ausonides* | 0.16 | -1.25 | 0.02 | 5.46 | 0.10 | |
| *Incisalia aug. iroides* | 0.16 | 0.03 | 0.03 | 5.77 | 0.10 | |
| *Vanessa virginiensis** | 0.15 | -0.36 | -0.01 | 9.00 | 0.18 | |
| *Plebejus acmon* | 0.15 | -0.14 | 0.06 | 9.62 | 0.49 | |
| *Adelpha bred. californica* | 0.15 | -0.23 | -0.03 | 6.58 | 0.21 | |
| *Boloria epithore* | 0.13 | -0.01 | -0.01 | 2.77 | 0.18 | |
| *Nymphalis californica** | 0.12 | 0.00 | -0.02 | 8.62 | 0.23 | |
| *Papilio rutulus* | 0.12 | 0.08 | 0.01 | 9.65 | 0.30 | |
| *Speyeria zerene* | 0.12 | 0.14 | -0.05 | 4.54 | 0.22 | |
| *Pieris rapae* | 0.11 | -0.32 | 0.03 | 9.77 | 0.72 | |
| *Euphilotes enoptes* | 0.11 | -0.30 | -0.08 | 3.04 | 0.16 | |
| *Plebejus saepiolus* | 0.10 | -0.17 | -0.01 | 3.85 | 0.30 | |
| *Plebejus lupini* | 0.10 | -0.08 | -0.05 | 3.08 | 0.21 | |
| *Strymon melinus* | 0.10 | -0.28 | 0.05 | 8.42 | 0.28 | |
| *Lycaena arota arota* | 0.09 | -0.23 | -0.13 | 3.81 | 0.10 | |
| *Battus philenor* | 0.09 | 0.22 | 0.00 | 5.15 | 0.23 | |
| *Satyrium saepium* | 0.09 | -0.16 | 0.00 | 4.85 | 0.13 | |
| *Glaucopsyche lygdamus* | 0.09 | -0.21 | 0.02 | 7.81 | 0.14 | |
| *Neophasia menapia* | 0.09 | -0.24 | -0.06 | 3.35 | 0.09 | |
| *Ochlodes sylvanoides* | 0.08 | -0.08 | -0.03 | 8.77 | 0.23 | |
| *Chlosyne palla* | 0.08 | -0.06 | -0.02 | 5.00 | 0.11 | |
| *Papilio zelicaon* | 0.07 | -0.52 | -0.01 | 9.04 | 0.28 | |
| *Limenitis lorquini* | 0.05 | -0.15 | -0.06 | 8.08 | 0.21 | |
| *Erynnis propertius* | 0.03 | -0.16 | 0.00 | 5.88 | 0.16 | |
| *Papilio multicaudatus* | 0.03 | -0.44 | 0.09 | 3.73 | 0.05 | |
| *Pontia sisymbrii* | 0.02 | -0.26 | 0.03 | 4.62 | 0.09 | |
| *Euchloe hyantis hyantis* | 0.00 | -0.22 | -0.02 | 3.00 | 0.18 | |
| *Parnassius clodius* | 0.00 | 0.07 | -0.06 | 4.00 | 0.33 | |
| *Satyrium californica* | -0.01 | -0.12 | 0.06 | 5.85 | 0.07 | |
| *Polites sabuleti sabuleti* | -0.02 | -1.22 | 0.01 | 3.88 | 0.18 | |
| *Satyrium sylvinus* | -0.02 | -0.50 | -0.06 | 6.69 | 0.10 | |
| *Apodemia mormo* | -0.02 | -0.36 | -0.06 | 2.19 | 0.07 | |
| *Incisalia eryphon* | -0.03 | 0.07 | -0.04 | 3.88 | 0.12 | |
| *Plebejus icarioides* | -0.05 | -0.15 | 0.02 | 5.69 | 0.20 | |
| *Lycaeides idas anna* | -0.11 | 0.00 | -0.11 | 3.19 | 0.23 | |

| Table S2: Results from maximum likelihood factor analysis of dispersal propensity variables from all butterfly species shown in Table S1 (including migrants). Two factors were generated using a varimax rotation and together characterized the natural history of focal taxa, including their dispersal propensity. For descriptions of how variables input into the factor analysis were calculated see *Methods*. | | |
| --- | --- | --- |
| *Input variables* | *Factor 1 loadings* | *Factor 2 loadings* |
| Elevational range | 0.465 | 0.243 |
| Diet Breadth | 0.697 | - |
| Geographical range | 0.706 | 0.495 |
| Wingspan | 0.12 | 0.531 |
|  | *Factor 1* | *Factor 2* |
| Variance explained | 0.30 | 0.15 |
| Cumulative variance explained | 0.30 | 0.45 |

| Table S3: Results from a maximum likelihood factor analysis of responses to weather from all non-migratory butterfly species shown in Table S1. Two factors were generated using a varimax rotation and together characterized the sensitivity to local climatic conditions. For descriptions of how variables input into the factor analysis were calculated see *Methods*. | | |
| --- | --- | --- |
| *Input variables* | *Factor 1 loadings* | *Factor 2 loadings* |
| Winter temperature | 0.218 | -0.4 |
| Spring temperature | 0.909 | 0.41 |
| Spring precipitation | 0.153 | 0.72 |
| Summer precipitation | -0.135 | -0.453 |
| Winter precipitation | -0.584 | - |
|  | *Factor 1* | *Factor 2* |
| Variance explained | 0.251 | 0.211 |
| Cumulative variance explained | 0.251 | 0.462 |

| Table S4: Results from a maximum likelihood factor analysis of responses to weather from all butterfly species shown in Table S1 (including migratory species). Two factors were generated using a varimax rotation and together characterized the sensitivity to local climatic conditions. For descriptions of how variables input into the factor analysis were calculated see *Methods*. | | |
| --- | --- | --- |
| *Input variables* | *Factor 1 loadings* | *Factor 2 loadings* |
| Winter temperature | -0.131 | 0.989 |
| Spring temperature | 0.996 | - |
| Spring precipitation | 0.534 | -0.177 |
| Summer precipitation | -0.239 | 0.16 |
| Winter precipitation | -0.216 | 0.141 |
|  | *Factor 1* | *Factor 2* |
| Variance explained | 0.28 | 0.21 |
| Cumulative variance explained | 0.28 | 0.49 |

| Table S5: Results from maximum likelihood factor analysis of natural history variables from all non-migratory butterfly species shown in Table S1. Two factors were generated using a varimax rotation and together characterized the natural history of focal taxa, including their dispersal propensity. For descriptions of how variables input into the factor analysis were calculated see *Methods*. | | |
| --- | --- | --- |
| *Input variables* | *Factor 1 loadings* | *Factor 2 loadings* |
| Elevational range | 0.36 | 0.311 |
| Host breadth | 0.572 | 0.549 |
| Geographical range | 0.797 | 0.292 |
| Wingspan | 0.175 | - |
|  | *Factor 1* | *Factor 2* |
| Variance explained | 0.274 | 0.129 |
| Cumulative variance explained | 0.274 | 0.403 |

| Table S6: Path coefficients and associated standard errors (SE) from a structural equation model examining the influence of natural history on variation in synchrony among butterfly species, including migrant taxa. For model structure see Fig. 2B. Model structure was well supported (χ^2^ = 1.41, *p =* 0.84, df = 4, n = 65; AIC = 714) and explained much of the variation in synchrony among taxa (*R*^2^ = 0.23). “Natural History 1” and “Natural History 2” refer to two factors extracted from a maximum likelihood factor analysis of species specific traits using the “psych” package in program R (see *Materials and Methods* for details and Table S8). These factors together characterized the natural history of a taxon, and are a proxy for dispersal propensity. “Decline” refers to the effect of year on fractional day positives. “Occupancy” refers to the average number of sites occupied by a given taxon each year averaged across all years. “Abundance” refers to the average fractional day positives of a given taxon across sites and years; it is a proxy for abundance. | | | |
| --- | --- | --- | --- |
| *Path (with migrants)* | *Coefficient* | *SE* | *p-value* |
| Dispersal 1 -> Synchrony | 0.516 | 0.133 | <0.001 |
| Dispersal 2 -> Synchrony | -0.034 | 0.13 | 0.792 |
| Abundance -> Synchrony | 0.044 | 0.139 | 0.753 |
| Occupancy -> Synchrony | -0.08 | 0.169 | 0.635 |
| Dispersal 1 -> Occupancy | 0.352 | 0.094 | <0.001 |
| Dispersal 2 -> Occupancy | 0.272 | 0.088 | 0.002 |
| Abundance -> Occupancy | 0.354 | 0.09 | <0.001 |
| Synchrony -> Decline | -0.232 | 0.117 | 0.048 |

| Table S7: Path coefficients and associated standard errors (SE) from a structural equation model examining the influence of natural history on variation in synchrony among butterfly species, excluding migrant taxa. For model structure see Fig. 2B. Model structure was well supported (χ ^2^ = 4.42, *p =* 0.35, df = 4, n = 56; AIC = 561) but did not explain much of the variation in synchrony among taxa (*R*^2^ = 0.03). “Natural History 1” and “Natural History 2” refer to two factors extracted from a maximum likelihood factor analysis of species specific traits using the “psych” package in program R (see *Materials and Methods* for details and Table S8). These factors together characterized the natural history of a taxon, and are a proxy for dispersal propensity. “Decline” refers to the effect of year on fractional day positives. “Occupancy” refers to the average number of sites occupied by a given taxon each year averaged across all years. “Abundance” refers to the average fractional day positives of a given taxon across sites and years; it is a proxy for abundance. | | | |
| --- | --- | --- | --- |
| *Path (without migrants)* | *Coefficient* | *SE* | *p-value* |
| Dispersal 1 -> Synchrony | -0.003 | 0.192 | 0.986 |
| Dispersal 2 -> Synchrony | 0.113 | 0.175 | 0.52 |
| Abundance -> Synchrony | 0.172 | 0.175 | 0.324 |
| Occupancy -> Synchrony | -0.12 | 0.188 | 0.525 |
| Dispersal 1 -> Occupancy | 0.177 | 0.133 | 0.182 |
| Dispersal 2 -> Occupancy | 0.3 | 0.116 | 0.01 |
| Abundance -> Occupancy | 0.409 | 0.107 | <0.001 |
| Synchrony -> Trend | -0.21 | 0.128 | 0.1 |

| Table S8: Path coefficients and associated standard errors (SE) from a structural equation model (SEM) examining the associations between sensitivity to climate and patterns of spatial synchrony among all butterfly species, including migratory taxa. For model structure see Fig. 2A. Model structure was well supported (χ^2^ = 5.41, *p =* 0.80, df = 9, n = 65, AIC=994) and explained much of the variation in synchrony among taxa (*R*^2^ = 0.50). “Climate 1” and “Climate 2” refer to two factors extracted from a maximum likelihood factor analysis of sensitivity to local climate using the “psych” package in program R (see *Materials and Methods* for details). These factors served as a proxy for the influence of local weather on synchrony. “ENSO” refers to the response to the sea-surface temperature anomaly (SSTA), which is indicative of the severity of the El Niño Southern Oscillation and is a proxy for response to regional, long-term climate patterns. “Decline” refers to the effect of year on fractional day positives. Occupancy” refers to the average number of sites occupied by a given taxon each year averaged across all years. “Abundance” refers to the average fractional day positives of a given taxon across sites and years. | | | |
| --- | --- | --- | --- |
| *Path (with migrants)* | *Coefficient* | *SE* | *p-value* |
| Climate 1 -> Synchrony | 0.288 | 0.092 | 0.002 |
| Climate 2 -> Synchrony | 0.153 | 0.093 | 0.098 |
| Occupancy -> Synchrony | -0.127 | 0.118 | 0.282 |
| ENSO -> Synchrony | 0.66 | 0.069 | <0.001 |
| Abundance -> Synchrony | 0.243 | 0.112 | 0.029 |
| ENSO -> Climate 2 | -0.32 | 0.108 | 0.003 |
| ENSO -> Climate 1 | -0.032 | 0.124 | 0.796 |
| Climate 1 -> Occupancy | 0.276 | 0.09 | 0.002 |
| Climate 2 -> Occupancy | -0.073 | 0.091 | 0.424 |
| Abundance - > Occupancy | 0.612 | 0.066 | <0.001 |
| Synchrony -> Decline | -0.235 | 0.117 | 0.044 |

| Table S9: Path coefficients and associated standard errors (SE) from a structural equation model examining the influence of climate on variation in synchrony among butterfly species, excluding migrant taxa. For model structure see Fig. 2A. Model structure was not supported (χ^2^ = 16.36, *p =* 0.06, df = 9, n = 56; AIC = 728) but explained much of the variation in synchrony among taxa (*R*^2^ = 0.28). “Climate 1” and “Climate 2” refer to two factors extracted from a maximum likelihood factor analysis of sensitivity to local climate using the “psych” package in program R (see *Materials and Methods* for details). These factors are a proxy for the influence of local weather on synchrony. “ENSO” refers to the response to the sea-surface temperature anomaly (SSTA), which is indicative of the severity of the El Niño Southern Oscillation and is a proxy for response to regional, long-term climate patterns. “Decline” refers to the effect of year on fractional day positives. “Occupancy” refers to the average number of sites occupied by a given taxon each year averaged across all years. “Abundance” refers to the average fractional day positives of a given taxon across sites and years; it is a proxy for abundance. | | | |
| --- | --- | --- | --- |
| *Path (without migrants)* | *Coefficient* | *SE* | *p-value* |
| Climate 1 -> Synchrony | 0.422 | 0.109 | <0.001 |
| Climate 2 -> Synchrony | -0.267 | 0.118 | 0.024 |
| Occupancy -> Synchrony | -0.144 | 0.154 | 0.35 |
| ENSO -> Synchrony | 0.037 | 0.119 | 0.755 |
| Abundance -> Synchrony | 0.205 | 0.148 | 0.165 |
| ENSO -> Climate 2 | -0.293 | 0.12 | 0.014 |
| ENSO -> Climate 1 | 0.023 | 0.134 | 0.866 |
| Climate 1 -> Occupancy | 0.191 | 0.098 | 0.051 |
| Climate 2 -> Occupancy | 0.151 | 0.098 | 0.124 |
| Abundance - > Occupancy | 0.633 | 0.069 | <0.001 |
| Synchrony -> Decline | -0.215 | 0.127 | 0.091 |

| Table S10: Path coefficients and associated standard errors (SE) from a structural equation model examining the combined influence of natural history and climate on variation in synchrony among butterfly species, including migratory taxa. For model structure see Fig. S1. Model structure was well supported (χ^2^ = 15.2, *p =* 0.65, df = 18, n = 65; AIC = 1219) and explained much of the variation in synchrony among taxa (*R*^2^ = 0.53). “Natural History 1” and “Natural History 2” refer to two factors extracted from a maximum likelihood factor analysis of species specific traits using the “psych” package in program R (see *Methods* for details and Table S9). These factors together characterize the natural history of a taxon, and are a proxy for dispersal propensity. “Climate 1” and “Climate 2” refer to two factors extracted from a maximum likelihood factor analysis of sensitivity to local climate and served as a proxy for the influence of local climate on synchrony. “ENSO” refers to the response to the sea-surface temperature anomaly, which is indicative of the severity of the El Niño Southern Oscillation and is a proxy for response to regional, long-term climate patterns. “Decline” refers to the effect of year on fractional day positives. “Occupancy” refers to the average number of sites occupied by a given taxon each year averaged across all years. “Abundance” refers to the average fractional day positives of a given taxon across sites and years; it is a proxy for abundance. | | | |
| --- | --- | --- | --- |
| *Path (with migrants)* | *Coefficient* | *SE* | *p-value* |
| Dispersal 1 -> Synchrony | 0.129 | 0.128 | 0.314 |
| Dispersal 2 -> Synchrony | -0.068 | 0.104 | 0.517 |
| Climate 1 -> Synchrony | 0.289 | 0.088 | 0.001 |
| Climate 2 -> Synchrony | 0.163 | 0.09 | 0.069 |
| ENSO -> Synchrony | 0.591 | 0.1 | <0.001 |
| Abundance -> Synchrony | 0.215 | 0.108 | 0.046 |
| Occupancy -> Synchrony | -0.144 | 0.132 | 0.275 |
| Dispersal 1 -> Occupancy | 0.352 | 0.094 | <0.001 |
| Dispersal 2 -> Occupancy | 0.272 | 0.088 | 0.002 |
| Abundance -> Occupancy | 0.354 | 0.09 | <0.001 |
| Dispersal 2 -> Climate 1 | 0.281 | 0.128 | 0.028 |
| Dispersal 1 -> Climate 1 | -0.007 | 0.132 | 0.958 |
| ENSO -> Climate 2 | -0.32 | 0.111 | 0.004 |
| Dispersal 1 -> ENSO | 0.593 | 0.08 | <0.001 |
| Synchrony -> Decline | -0.24 | 0.117 | 0.04 |

| Table S11: Path coefficients and associated standard errors (SE) from a structural equation model examining the combined influence of natural history and climate on variation in synchrony among butterfly species, excluding migrant taxa. For model structure see Fig. S1. Model structure was not supported (χ ^2^ = 36.1, *p =* 0.007, df = 18, n = 56; AIC = 911) but explained much of the variation in synchrony among taxa (*R*^2^ = 0.37). “Natural History 1” and “Natural History 2” refer to two factors extracted from a maximum likelihood factor analysis of species specific traits using the “psych” package in program R (see *Materials and Methods* for details). These factors together characterize the natural history of a taxon, and are a proxy for dispersal propensity. “Climate 1” and “Climate 2” refer to two factors extracted from a maximum likelihood factor analysis of sensitivity to local climate and served as a proxy for the influence of local climate on synchrony. “ENSO” refers to the response to the sea-surface temperature anomaly, which is indicative of the severity of the El Niño Southern Oscillation and is a proxy for response to regional, long-term climate patterns. “Decline” refers to the effect of year on fractional day positives. “Occupancy” refers to the average number of sites occupied by a given taxon each year averaged across all years. “Abundance” refers to the average fractional day positives of a given taxon across sites and years; it is a proxy for abundance. | | | |
| --- | --- | --- | --- |
| *Path (without migrants)* | *Coefficient* | *SE* | *p-value* |
| Dispersal 1 -> Synchrony | 0.019 | 0.161 | 0.908 |
| Dispersal 2 -> Synchrony | 0.265 | 0.141 | 0.061 |
| Climate 1 -> Synchrony | 0.463 | 0.097 | <0.001 |
| Climate 2 -> Synchrony | -0.321 | 0.107 | 0.003 |
| ENSO -> Synchrony | -0.053 | 0.12 | 0.655 |
| Abundance -> Synchrony | 0.172 | 0.141 | 0.221 |
| Occupancy -> Synchrony | -0.256 | 0.151 | 0.09 |
| Dispersal 1 -> Occupancy | 0.177 | 0.133 | 0.182 |
| Dispersal 2 -> Occupancy | 0.3 | 0.116 | 0.01 |
| Abundance -> Occupancy | 0.409 | 0.107 | <0.001 |
| Dispersal 2 -> Climate 1 | -0.149 | 0.167 | 0.372 |
| Dispersal 1 -> Climate 1 | 0.106 | 0.167 | 0.524 |
| ENSO -> Climate 2 | -0.293 | 0.122 | 0.017 |
| Dispersal 1 -> ENSO | 0.396 | 0.113 | <0.001 |
| Synchrony -> Decline | -0.223 | 0.127 | 0.078 |
